# Identification of a novel *GNAS* mutation in a family with pseudohypoparathyroidism type 1A

**DOI:** 10.1186/s12887-024-04761-8

**Published:** 2024-04-25

**Authors:** Fabio Sippelli, Silvana Briuglia, Chiara Ferraloro, Anna Paola Capra, Emanuele Agolini, Tiziana Abbate, Giorgia Pepe, Tommaso Aversa, Malgorzata Wasniewska, Domenico Corica

**Affiliations:** 1 Department of Human Pathology of Adulthood and Childhood, University of Messina, Messina, Italy; 2 Department of Biomedical and Dental Sciences and Morphofunctional Imaging, University of Messina, Messina, Italy; 3 Department of Chemical, Biological, Pharmaceutical, and Environmental Sciences, University of Messina, Messina, Italy; 4 Translational Cytogenomics Research Unit, Bambino Gesù Children’s Hospital, IRCCS, Rome, Italy

**Keywords:** PHP1A, Albright hereditary osteodystrophy, PTH resistance, *GNAS* mutation, Genotype–phenotype variability

## Abstract

**Background:**

Pseudohypoparathyroidism (PHP) is caused by loss-of-function mutations at the *GNAS* gene (as in the PHP type 1A; PHP1A), de novo or inherited at heterozygous state, or by epigenetic alterations at the *GNAS* locus (as in the PHP1B). The condition of PHP refers to a heterogeneous group of disorders that share common clinical and biological features of PTH resistance. Manifestations related to resistance to other hormones are also reported in many patients with PHP, in association with the phenotypic picture of Albright hereditary osteodystrophy characterized by short stature, round facies, subcutaneous ossifications, brachydactyly, mental retardation and, in some subtypes, obesity. The purpose of our study is to report a new mutation in the *GNAS* gene and to describe the significant phenotypic variability of three sisters with PHP1A bearing the same mutation.

**Case presentation:**

We describe the cases of three sisters with PHP1A bearing the same mutation but characterized by a significantly different phenotypic picture at onset and during follow-up in terms of clinical features, auxological pattern and biochemical changes. Clinical exome sequencing revealed a never before described heterozygote mutation in the *GNAS* gene (NM_000516.5 c.118_139 + 51del) of autosomal dominant maternal transmission in the three siblings, confirming the diagnosis of PHP1A.

**Conclusions:**

This study reported on a novel mutation of *GNAS* gene and highlighted the clinical heterogeneity of PHP1A characterized by wide genotype–phenotype variability. The appropriate diagnosis has crucial implications for patient care and long-term multidisciplinary follow-up.

## Background

The term pseudohypoparathyroidism (PHP) refers to a spectrum of genetic disorders characterized by clinical and biological features of resistance to parathyroid hormone (PTH) [1]. PHP is caused by loss-of-function mutations at the *GNAS* gene, de novo or inherited at heterozygous state, or by epigenetic alterations at the *GNAS* locus [1, 2]. *GNAS* gene, located on chromosome 20q13, encodes the α-subunit of the stimulatory G protein (Gsα) [1]. Manifestations related to resistance to other protein hormones, which act by binding to Gsα‐protein coupled receptors, such as TSH, gonadotropins (FSH/LH) and to lesser extent growth hormone (GH), are also reported in patients with PHP, in association with the phenotypic picture of Albright hereditary osteodystrophy (AHO) characterized by short stature, round facies, subcutaneous ossifications, and brachydactyly and varying degrees of cognitive impairment; obesity is present in some subtypes [2–4].

Based on the type of *GNAS* defect, five subtypes of PHP can be distinguished: type 1A (PHP1A, maternal inactivating mutations or de novo mutations) OMIM#103580; type 1B (PHP1B, imprinting dysregulation) OMIM#603233; type 1C (PHP1C, few inactivating mutations at the last amino acids reported) OMIM#612462; pseudopseudohypoparathyroidism (PPHP) OMIM#612463 and progressive osseous heteroplasia (POH) OMIM#166350 (both characterized by paternal inactivating mutations or de novo mutations). Patients with PHP1A and PHP1C show variable expression of phenotypic features of AHO [2, 5]. In PHP1B, patients typically manifest no evidence of AHO but show exclusive hormonal resistance to PTH and TSH [6]. In a subgroup of patients with PHP and various features of AHO, epigenetic mutations of *GNAS* similar to those classically detected in PHP1B patients were identified, suggesting a genetic overlie between PHP1A and PHP1B [7–11]. Patients with PPHP show the classical clinical features of AHO, in the absence of PTH resistance; only in a few cases there is mild resistance to PTH and TSH [5]. POH is distinguished by the presence of ectopic ossifications with a progressive extension into connective tissue. Patients with POH usually have no other features of AHO and have normal responsiveness to PTH [5].

The exact prevalence of PHP and related disorders is unknown. A prevalence of PHP ranging from 0.34 per 100.000 to 1.1 per 100.000 is described in the literature, but these data are often biased by the lack of genetic diagnostic confirmation [5]. Clinical presentation and disease severity can vary considerably between patients, even in the same family and among individuals carrying the same genetic alteration. In literature, few descriptions of PHP1A affected siblings with some minor clinical and hormonal differences despite having the same mutation are reported [12–17].

The main criteria proposed for the clinical diagnosis of PHP or related disorders include PTH resistance, ectopic ossifications and brachydactyly. The clinical diagnosis requires the presence of at least one of the major criteria between PTH resistance and ectopic ossification, or the presence of brachydactyly and at least two minor criteria, including TSH resistance, other hormonal resistance, motor and cognitive retardation or impairment, intrauterine and postnatal growth retardation, obesity, flat nasal bridge and/or maxillary hypoplasia and/or round facies [18].

To date, more than 180 different mutations in the *GNAS* that lead to PHP1A are known [19]. At the same time, two-thirds of these mutations are found only once time. There is no strict correlation between the genotype and phenotype in *GNAS* variants. The GNAS locus has a complex structure and variable expression that conditions clinical variability [2], so a wide variability in both phenotype and severity of progression are described [19, 20].

In the present study, we report a novel pathogenic mutation in the *GNAS* gene identified in a PHP1A family cluster and we describe the significant phenotypic variability of three sisters with PHP1A bearing the same mutation.

### Case presentation

We describe the cases of three sisters with PHP1A bearing the same mutation but characterized by a significantly different phenotypic picture at first evaluation and during follow-up in terms of clinical and auxological features, and biochemical findings. The maternally inherited *GNAS* gene mutation of these three sisters is reported here for the first time in scientific literature.

**Fig. 1:**
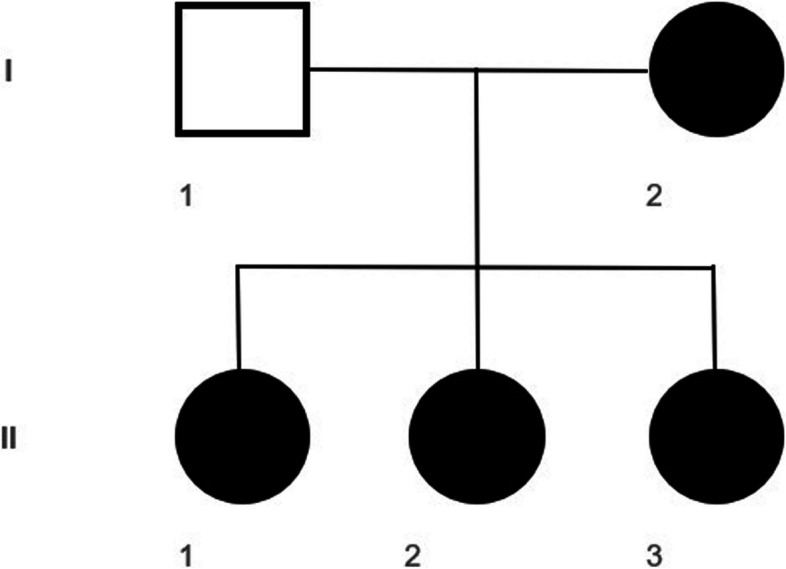
Pedigree of the described family. The open symbols indicate pedigree members without PHP1A; the full black symbols, members with PHP1A

The mother's phenotype was characterized by the presence of mild signs of PHP1A such as disproportionate short stature, brachydactyly, round face and subcutaneous ossifications, which were significantly milder than those manifested by her daughters. She also reported a history of hypocalcemia at a young age and transient subclinical hypothyroidism. The mother reported no drug therapy. We had no data concerning any hormonal resistance of the mother as she had not investigated these aspects and was not being cared for at an endocrinological Centre. Pedigree of the family is shown in Figure 1.

#### Genetic

In order to define the diagnosis and provide family counseling, the patient (II:1) was referred for genetic testing (Fig. 1).

Clinical Exome Sequencing (CES) was performed with clinical indications and included the proband (II:1) plus the family members, two sisters (II:2 and II:3) and their parents (I:1 and I:2) after written informed consent was provided.

Enrichment and parallel sequencing were performed on genomic DNA extracted from circulating leukocytes of all the subjects. Library preparation was carried out using a Twist Custom Panel (clinical exome - Twist Bioscience) according to manufacturer’s protocol and sequenced on a NovaSeq6000 (Illumina).

Bioinformatic analysis was performed through the BWA Aligner or DRAGEN Germline Pipeline systems and the sequences were aligned to the reference human genome GRCh37. Geneyx Analysis software (Knowledge-Driven NGS Analysis tool powered by the GeneCards Suite) was used for filtering and variant calling.

Based on the guidelines of the American College of Medical Genetics and Genomics, a minimum depth coverage of 30X was considered suitable for analysis. Variants were examined for coverage and Q score (minimum threshold of 30) and visualized by the Integrative Genome Viewer (IGV). Functional impact of variants was analyzed by Combined Annotation-dependent Depletion (CADD) V.1.3, Sorting Intolerant from Tolerant (SIFT) and Polymorphism Phenotyping v2 (PolyPhen-2). Rare variants (MAF < 0.1%) were filtered according to the frequency database of the general population (gnomAD).

The analysis identified a novel intronic variant in the *GNAS* gene (NM_000516.5) in the three sisters and in their mother (c.118_139+51del). The effect of this sequence alteration is predicted to disrupt the canonical splice site that flanks exons. This variant results not previously reported and it is not present in the gnomAD database. For the interpretation in the clinical setting, according to the ACMG guidelines, this variant can be classified as a likely pathogenic variant (class 4) [21, 22].

Genetic diagnoses were made at ages 11 for subject II:1, 9 for subject II:2 and 4 for subject II:3 (Fig. 1).

#### Clinical features

**Fig. 2:**
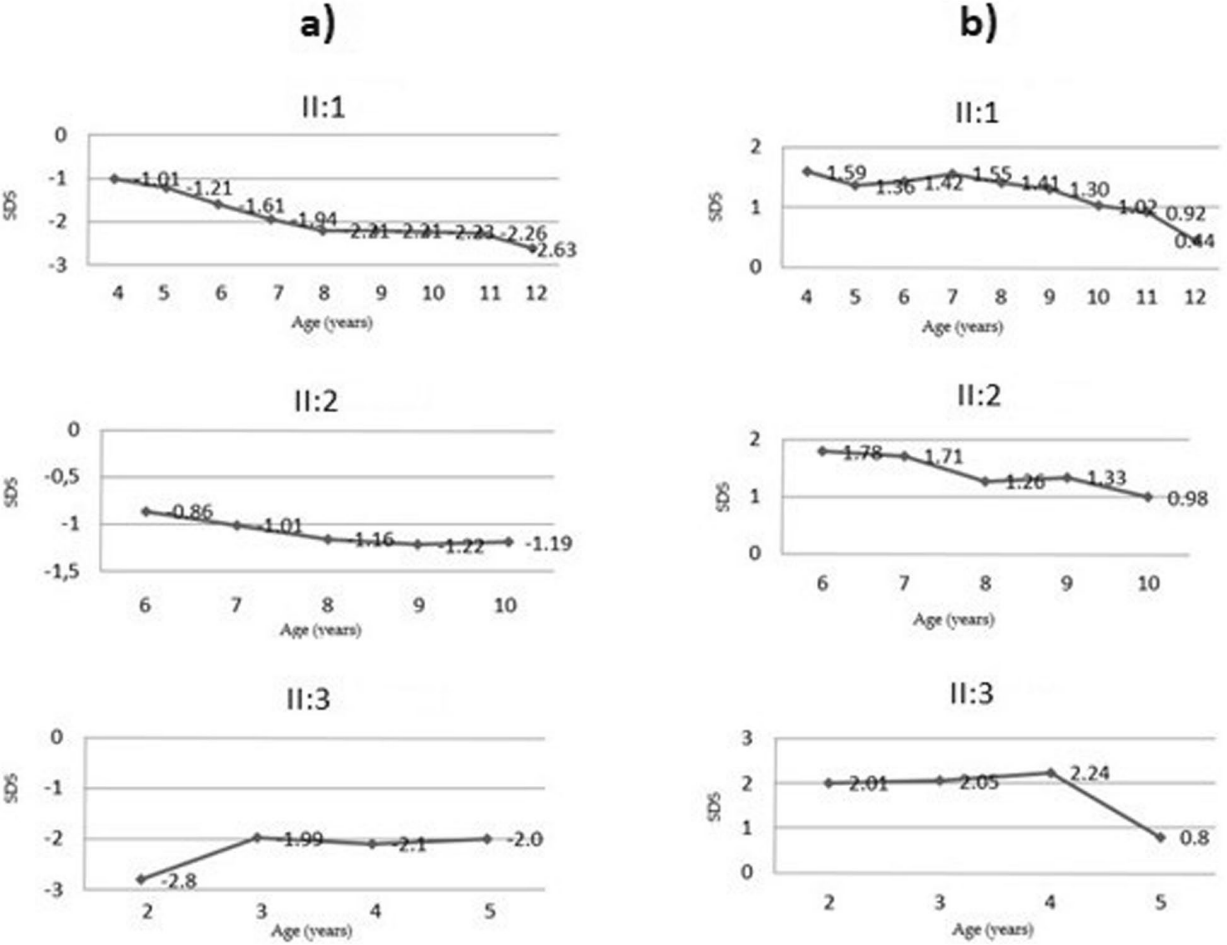
Growth charts of reported patients. **a** Growth charts of height SDS with respect to age (**b**) Growth charts of BMI SDS with respect to age

From the auxological point of view, the subject II:1, who came to our observation at the age of 4 years old for the occasional finding of hypocalcemia and hyperparathyroidism, had a clinical picture characterized by progressive impaired height growth and obesity documented during the 8-year follow-up (Fig. 2). The subject II:2, brought to our observation at the age of 6 years old for the finding of hypothyroidism on control hematological investigations, by contrast had quite regular stature growth (between 10th and 20th centile for age according to the WHO growth chart) during 4 years of follow-up and obesity (Fig. 2). The subject II:3, who came to our observation at the age of 2 years old for caring in patient with congenital hypothyroidism and following our indication when suspicion of PHP was advanced for the other two sisters, had significant short stature and obesity from early childhood (Fig. 2). Before coming to our attention, the patients had not been on vitamin D nor calcium therapy.

Over the course of follow-up, the three patients presented a progressive reduction of their overweight, which could be attributed, at least in part, to the dietary-behavioral counseling carried out during the visits.

**Fig. 3:**
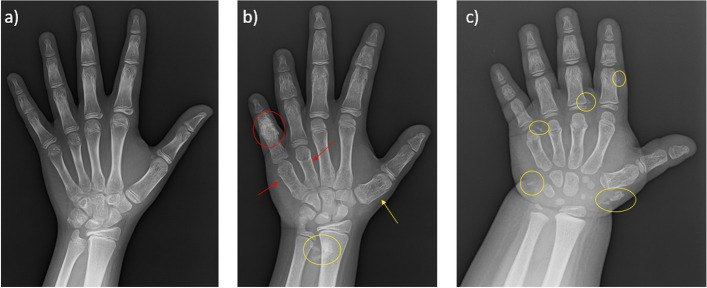
Bone age of reported patients. **a** Left hand and wrist x-ray of the patient II:1 without significant bone alterations. **b** Left hand and wrist x-ray of the patient II:2. Brachydactyly of the IV and V metacarpus (red darts). Osteopenia and swelling of the metacarpal of the first finger (yellow dart). Osteosclerosis of the proximal phalanx of the V ray with disappearance of the proximal interphalangeal space (red circle). Large soft tissue ossifications close to the distal radius and ulna epiphysis (yellow circle). **c** Left hand and wrist x-ray of the patient II:3. ossifications in the context of soft tissues of I, II and V finger (yellow circle)

With regard to clinical features, described according to Phenomizer [23, 24], each of the three sisters showed a variable expression of AHO. In detail, subject II:1 had the following characteristics of AHO: short stature, round face, subcutaneous ossifications in the right breast region and left thigh and mild global developmental delay, associated with obesity. Subject II:2 had a round face, obesity, brachydactyly with short 4th and 5th metacarpal, subcutaneous ossifications in the soft tissues of hands and feet, and mild global developmental delay. Subject II:3 had a round face, subcutaneous ossifications on hands and feet, and delayed speech and language development but she did not yet show overt brachydactyly. Significant differences were also documented with respect to bone alterations identifiable on the left wrist and hand X-ray as shown in Figure 3.

Moreover, patient II:1 had regularly initiated development of puberty with the appearance of thelarche at age 10 years, but during follow-up over 21 months there was no progression of thelarche or any other features of puberty elements, which subsequently led to a diagnosis of hypogonadism and the need to start estrogen replacement therapy.

With regard to hormonal patterns, the three patients showed considerable differences from each other. At the time of diagnosis, all sisters presented hyperparathyroidism, but only patient II:1 had hypocalcemia and only patient II:2 and patient II:3 showed hyperphosphatemia (Table 1). Patients II:1 and II:3 had congenital hypothyroidism (diagnosed with neonatal screening and therefore on levothyroxine therapy from the earliest ages of life), instead patients II:2 developed hypothyroidism at the age of 6 years old. Furthermore, all three sisters had basal GH and IGF-1 levels within normal limits for age. Moreover, no alterations in glucose metabolism were detected in any of the three sisters. Data on the phenotype of our patients are presented in Table 1.

**Table 1:** Clinical and biochemical characteristics of the three sisters at first evaluation and during follow-up

	**II:1**	**II:2**		**II:3**	**Normal values**
Age at clinical diagnosis	4 years old	6 years old		2 years old	n.a
Growth abnormality^a^	Short stature	No		Childhood onset short stature	n.a
Overweight/Obesity	Obesity	Obesity		Obesity	n.a
Rounded face	Yes	Yes		Yes	n.a
Brachydactyly	No	Yes		No	n.a
Subcutaneous ossifications	Yes	Yes		Yes	n.a.
Global developmental delay	mild	mild		Delayed speech	n.a
Hypothyroidism	Congenital	Acquired		Congenital	n.a
PTH (pg/ml)	714	783		511.2	15 – 65
Calcium (mg/dl)	7.4	8.9		8.8	8.2 – 10.4
Phosphorus (mg/dl)	4.5	7.1		6.0	2.5 – 4.6

^a^Growth pattern was assessed during follow-up, whereas the other data in the table are from each patient's first visit

(n.a.) not appropriate

## Discussion and conclusions

PHP is a highly heterogeneous hereditary disorder and its clinical presentation, evolution and complications reflect wide genotype–phenotype variability.

In these case reports, we describe a significant clinical-auxological and biochemical variability at time of diagnosis and during follow-up in three sisters affected by PHP1A with a novel maternally inherited *GNAS* mutation. Molecular genetic diagnosis allowed us to detect the specific PHP variants in the 3 siblings, bearing a non-previously documented splicing variant in the *GNAS* gene, c.[118_139 + 51del];[ =] p.[(?)];[( =)]. PHP1A is caused by inactivating variants on the maternal allele of the *GNAS* gene.

The gene variant detected in our cases determines the loss of a canonical splice site, resulting in the loss of the correct protein-coding sequence, so it can be considered pathogenic and therefore the cause of the disease.

A similar deletion, c.119_139 + 17del, was previously described in another family and it is reported in ClinVar as pathogenic, resulting in a splice junction loss with the predicted formation of an inactive protein with an altered carboxyl-terminus [25].

Multiple previously reported disease-associated variants appear to localize in the intronic regions of *GNAS* gene near splicing sites. It was shown that these variants frequently lead to aberrant splicing, including the present cases and previously reported cases [20]. The *GNAS* variant within an intron identified in our cases might be overlooked or difficult to interpret in conventional genetic screening tests, considering the variant type, never before reported, and location in a non-coding region. The mother was shown to be the carrier of the variant, which agrees with the established inheritance of the disease. The effect of this mutation on transcribed mRNA has not been tested; however, although it cannot directly prove that the mutation induces haploinsufficiency in the functional Gsα product, the clinical phenotype and typical pattern of inheritance support the pathogenicity of this novel intronic variant.

There is a 50% possibility of transmitting this genetic variant, and the descendant will develop PPHP or POH (if the affected subject is male) or PHP1A or PHP1C (if the affected subject is female, as in the described cases). In the case of pathogenetic variants with maternal segregation, all these clinical characteristics can manifest with varying degrees.

As observed, the same mutations could show interfamilial and intrafamilial variability, and the effect of imprinting on the *GNAS* gene might also reflect a spectrum of clinical outcomes. This variability is related to the imprinting expression of biallelic heredity. Upstream of the *GNAS* gene there are several regions subject to genomic imprinting, an epigenetic phenomenon whereby the transcription of an allele depends on its parental origin [2]. On the other hand, *GNAS* gene expression is subject to tissue-dependent genomic imprinting: in most cells, gene expression is biallelic but in some hormone-responsive tissues, such as thyroid, pituitary, gonads, and renal proximal tubules, the gene is subject to paternal genomic imprinting, i.e., transcription and synthesis of the Gsα protein occurs mostly from the maternal chromosome (this is therefore referred to as “partial paternal imprinting” or “preferential maternal expression”) [2, 26]. The progressive course of the onset of some clinical manifestations of PHP, such as PTH resistance at renal proximal tubules, could be explained by these mechanisms, although the mechanisms are not entirely clear [2, 26].

In consideration of the wide phenotypic variability of PHP and the overlapping clinical and biochemical features among the different forms, in 2016 the EuroPHP Network proposed a new classification for disorders related to the alteration of the PTH response pathway mediated by Gsα protein activation [18]. They agreed on the use of the term ‘inactivating PTH/PTHrP signaling disorder’ (iPPSD) for those patients who have at least one of the major criteria including PTH resistance, ectopic ossifications and brachydactyly. The novel classification of iPPSD is based on the presence of the pathophysiology of the PTH/PTHrP signaling abnormalities while the number of the subtype iPPSD is based on the underlying molecular (epi-) genetic defect (responsible for the pathology) [18].

To date, no correlation can be highlighted taking into consideration type or location of *GNAS* mutations and disease onset, severity of endocrine resistance, neurocognitive phenotype, or degree of AHO-related alterations. Genetic counseling is essential for the classification of these patients, although the specific mutation cannot always be documented with currently available testing techniques, like PCR-based sequencing. In these cases, submicroscopic *GNAS* deletion [12] or epigenetic alterations such as methylation defects of *GNAS* locus, must be suspected, and multiplex ligation-dependent probe amplification (MLPA) or comparative genomic hybridization arrays (aCGH) must be performed [5].

A literature review was performed from which we extrapolated only six case reports of families with PHP1A with more than one family member with the same genotype (each family presented two affected children carrying the same variant) [12–17] (Table 2). By comparing affected individuals belonging to the same family with each other, it was possible to document differences regarding age at disease diagnosis, phenotypic traits, auxological features, biochemical alterations of calcium-phosphorus metabolism and hormonal resistance, despite having the same genotype (Table 2). Analyzing the phenotype of the reported cases, there is no evident sexual dimorphism in the expression of the disease. No details regarding hormonal resistance to GH and gonadotropins and the presence of bone alterations are described in these case reports.

**Table 2:** Phenotype and biochemical-hormonal features of siblings with PHP1A with *GNAS* inactivating mutation described in literature

	**AD**	**S**	**SS**	**OB**	**RF**	**BR**	**SO**	**CI**	**PTH**	**Ca**	**P**	**TSH**	**GH**	**FSH-LH**	**Mutation**
**Mitsui et al. **[12]															
** Patient 1**	10	F	Y	Y	Y	Y	Y	No	Hi	N	Hi	Hi	N	N	Deletion of whole gene, *mat*
** Patient 2**	10	M	Y	No	Y	No	Y	No	Hi	Lo	Hi	Hi	N	N	Deletion of whole gene, *mat*
**Wang et al. **[13]															
** Patient 1**	7	M	Y	Y	Y	Y	Y	Un	Hi	Lo	Hi	Hi	Un	Un	c.277C > T in exon 4 p.(Gln93Ter), de novo
** Patient 2**	5	M	No	Y	No	Y	Y	Un	Hi	N	Hi	N	Un	Un	c.277C > T in exon 4 p.(Gln93Ter), de novo
**Pinsker et al. **[14]															
** Patient 1**	2	F	Y	Y	Un	Y	Y	Y	Un	Lo	Un	Hi	Un	Un	c.305C > A in exon 4 p.(Ala102Glu), *mat*
** Patient 2**	1	F	Y	No	No	Y	Y	Y	Hi	Lo	Un	Hi	Un	Un	c.305C > A in exon 4 p.(Ala102Glu), *mat*
**Walden et al. **[15]															
** Patient 1**	3	F	Y	Y	Y	Y	Y	Y	Hi	Lo	Hi	Hi	Un	Un	4 bp deletion in exon 7, *mat*
** Patient 2**	4	M	Y	Y	Y	Y	Y	No	Hi	Lo	Hi	Hi	Un	Un	4 bp deletion in exon 7, *mat*
**Kayemba-Kay’s et al. **[16]															
** Patient 6**	4	M	Un	Y	No	Y	No	Y	Hi	N	N	Hi	Lo	Un	c.344C > T in exon 5 p.(Pro115Leu), *mat*
** Patient 7**	7	F	Un	Y	Y	No	No	No	N	N	N	Hi	Un	Un	c.344C > T in exon 5 p.(Pro115Leu), *mat*
**Han et al. **[17]															
** Patient 6**	9	M	Y	Y	Y	Y	Y	Y	Hi	N	N	Hi	N	Un	c.348dup in exon 5 p.(Val117fs), *mat*
** Patient 7**	9	M	No	Y	Y	Y	No	Y	N	N	N	Hi	Un	Un	c.348dup in exon 5 p.(Val117fs), *mat*

*AD* Age at diagnosis (years), *Y *yes, *S *Sex, *SS* Short stature, *OB *Obesity, *RF *Round face, *BR *Brachydactyly,  *SO *Subcutaneous ossifications, *CI* Cognitive impairment, *Hi *High, *Lo *low, *N *Normal, *Un *Unknown

Therefore, the genotype–phenotype variability highlighted by our case series seems to be evident by analyzing the data of familial cases with PHP1A with the *GNAS* inactivating mutation found in scientific literature. Analyzing the early clinical features of our patients and those reported in literature regarding siblings with PHP1A (Table 2), among the most frequent clinical presenting features, those that can guide us for early diagnosis are: obesity (86%), brachydactyly (80%), subcutaneous ossifications (80%) and short stature (76%), while congenital or acquired hypothyroidism is the most frequent biochemical feature (93%). These data confirm what was previously reported by Mantovani et al. [5]. Fisher et al. [25] described another series of familial cases with PHP1A characterized by a high incidence of elevated TSH (5 out of 6 cases) and hypocalcaemia (4 out of 6 cases). However, these authors reported insufficient clinical and biochemical information to describe the disease phenotype and therefore insufficient to be compared with the other familial cases analyzed in our literature review.

In conclusion, the cases described in the present study, bearing a maternally inherited *GNAS* mutation described here for the first time, confirm the wide phenotypic variability of PHP1A at onset and during follow-up. Moreover, this case series allows us to emphasize the recommendation to promptly investigate the family nucleus when a PHP case is identified, in order to limit the diagnostic delay. For this aspect, genetic counseling plays a crucial role. An early and multidisciplinary approach to the individual with PHP is crucial with respect to the outcomes and long-term follow-up of this condition [27]. Future studies on larger case series will be needed to further investigate the genotype–phenotype correlation in this heterogeneous group of inherited disorders, since only through a thorough understanding of the characteristics of these rare conditions it will be possible to promote early diagnosis and improve the clinical-therapeutic management of these patients.

## Data Availability

The datasets used and/or analyzed during the current study are available from the corresponding author on reasonable request.

## Abbreviations

PHP: Pseudohypoparathyroidism
PHP1A: Pseudohypoparathyroidism type 1A
PHP1B: Pseudohypoparathyroidism type 1B
PHP1C: Pseudohypoparathyroidism type 1C
PTH: Parathyroid hormone
PPHP: Pseudopseudohypoparathyroidism
POH: Progressive osseous heteroplasia
AHO: Albright hereditary osteodystrophy
TSH: Thyroid stimulating hormone
CES: Clinical exome sequencing
MLPA: Multiplex ligation-dependent probe amplification
aCGH: Comparative genomic hybridization arrays
